# Lack of toilets and safe water in health-care facilities

**DOI:** 10.2471/BLT.15.154609

**Published:** 2015-04-01

**Authors:** Jamie Bartram, Ryan Cronk, Maggie Montgomery, Bruce Gordon, Maria Neira, Edward Kelley, Yael Velleman

**Affiliations:** a Water Institute and Department of Environmental Sciences and Engineering, University of North Carolina, Chapel Hill, North Carolina, 27599, United States of America.; b Department of Public Health, Environmental and Social Determinants of Health, World Health Organization, Geneva, Switzerland.; c Department of Service Delivery and Safety, World Health Organization, Geneva, Switzerland.; d WaterAid, London, England.

In March 2015, the World Health Organization (WHO) and the United Nations Children’s Fund (UNICEF) released a report^1^ on the status of water and sanitation in health-care facilities from 54 low- and middle-income countries. Data representing 66 000 health facilities show that water was not readily available in about 40%.^1^ Over a third of facilities lacked soap for hand washing and a fifth lacked toilets. In many countries, in facilities where water is available, there is no guarantee that it is safe for consumption.^2^

This is a major embarrassment for the health sector: health facilities serve as foci for infection and patients seeking treatment fall ill and may die, for the lack of the most basic requirements for good hygiene – safe, reliable water supplies and adequate sanitation. Pregnant mothers rely on a birthing environment that, at a minimum, does not place them or their baby at risk. Infections cause nearly half of late neonatal deaths (430 000)^3^ – many of which are attributable to inadequate hygiene. The same conditions contribute to major disease outbreaks, such as cholera, as well as the spread of antimicrobial resistance – another major public health threat.^4^

Safe water and adequate sanitation are fundamental to a healthy and dignified life. The benefits of water and sanitation include diarrhoeal diseases averted, other infections prevented, better nutrition, financial and economic savings, and improved education, especially for girls.^5^ Proposals for the Sustainable Development Goals include a target to achieve universal access to basic drinking water, sanitation and hygiene for households, schools and health-care facilities, by 2030.

Improved hygiene in health-care facilities is an urgent need and a strategic investment for health objectives, including maternal and child health, infection prevention, outbreak response and health systems strengthening.^6^

First, policies and standards should be established and progress tracked on delivering appropriate services. Countries with national plans and targets have better water and sanitation in health-care facilities.^1^ This suggests that national planning can drive service expansion. Guidelines, such as WHO’s *Essential environmental health standards in health care*, can assist in policy-making, including standards appropriate for different circumstances.^7^ Existing monitoring initiatives, such as health management information systems could readily incorporate water, sanitation and hygiene indicators. Service availability and readiness assessments,^1^ enable comparison between facilities (for benchmarking); between countries and facility types (for resource allocation and standard setting); and over time (to monitor progress).

Second, sufficient human and financial resources, along with systems to support service delivery, are required. Service improvements would benefit from comprehensive, facility-based risk assessments and associated risk management plans.^8^ Such plans, and the inputs required to manage them, need to be embedded in infection prevention and control efforts and in health system funding mechanisms. The Clean and Safe Health Facility Campaign in Ethiopia is a successful example.^9^ This campaign aims to reduce infections in health-care settings, through staff training, improved water, sanitation, clinical waste management and facility auditing. 

Third, better leadership and coordination are needed for closely related health and development initiatives such as universal health coverage,^10^ maternal and child health, infection control and energy access. Leveraging the obligation of states to progressively realize the human right to water and sanitation provides another means to accelerate and recognize action.^11^ However, leadership within the health sector alone is insufficient. While some aspects, such as hand washing by health-care providers, fall within the remit of the health system; others, such as safe, reliable water supplies and functional sanitation systems, extend beyond it. Progress demands inter-sectoral action, using the expertise of health professionals and engaging local and central governments as well as civil society.

The WHO/UNICEF report portrays a hidden, but remediable, health crisis. With international leadership from WHO and commitments from governments and development partners, rather than serving as foci for harm, health facilities should and can become models of clean, safe and dignified care.
